# Supplementary material for: Aberrant sorting of hippocampal complex pyramidal cells in type I lissencephaly alters topological innervation
Source: eLife. 2020 Jun 19;9:e55173. doi: 10.7554/eLife.55173 (PMC7340499; doi:10.7554/eLife.55173)
Supplement: Transparent reporting form [file elife-55173-transrepform.docx]

***eLife’s* transparent reporting form**

We encourage authors to provide detailed information *within their submission* to facilitate the interpretation and replication of experiments. Authors can upload supporting documentation to indicate the use of appropriate reporting guidelines for health-related research (see [EQUATOR Network](http://www.equator-network.org/%20)), life science research (see the [BioSharing Information Resource](https://biosharing.org/)), or the [ARRIVE guidelines](http://www.plosbiology.org/article/info:doi/10.1371/journal.pbio.1000412) for reporting work involving animal research. Where applicable, authors should refer to any relevant reporting standards documents in this form.

If you have any questions, please consult our Journal Policies and/or contact us: [editorial@elifesciences.org](mailto:editorial@elifesciences.org).

**Sample-size estimation**

- You should state whether an appropriate sample size was computed when the study was being designed
- You should state the statistical method of sample size computation and any required assumptions
- If no explicit power analysis was used, you should describe how you decided what sample (replicate) size (number) to use

Please outline where this information can be found within the submission (e.g., sections or figure legends), or explain why this information doesn’t apply to your submission:

We did not pre-determine sample sizes prior to the outset of the study. As a general guideline in immunohistochemical experiments, we aim for 5-12 slices per group to be analyzed – from 4 or more animals from 2-3 litters. This information is in figure legends, associated text, and provided data sheets.

**Replicates**

- You should report how often each experiment was performed
- You should include a definition of biological versus technical replication
- The data obtained should be provided and sufficient information should be provided to indicate the number of independent biological and/or technical replicates
- If you encountered any outliers, you should describe how these were handled
- Criteria for exclusion/inclusion of data should be clearly stated
- High-throughput sequence data should be uploaded before submission, with a private link for reviewers provided (these are available from both GEO and ArrayExpress)

Please outline where this information can be found within the submission (e.g., sections or figure legends), or explain why this information doesn’t apply to your submission:

Replicates are listed in figure legends. Almost all bar graphs show summary values and individual data points. N’s for each experiment are described in text and legends of the associated figures as either cell number, slice number or animal number. For monosynaptic stimulation experiments, principal cells that showed no baseline response, or in disynaptic experiments, no reduction of the disynaptic IPSC with application of excitatory transmission blockers, were excluded – this is explained in the methods. Outliers were not excluded. Note that some graphs are shown zoomed to highlight the distribution of the bulk of the points and do not show 1 or 2 points outside the y-axis range. These points are listed in the provided dataset sheets.

**Statistical reporting**

- Statistical analysis methods should be described and justified
- Raw data should be presented in figures whenever informative to do so (typically when N per group is less than 10)
- For each experiment, you should identify the statistical tests used, exact values of N, definitions of center, methods of multiple test correction, and dispersion and precision measures (e.g., mean, median, SD, SEM, confidence intervals; and, for the major substantive results, a measure of effect size (e.g., Pearson's r, Cohen's d)
- Report exact p-values wherever possible alongside the summary statistics and 95% confidence intervals. These should be reported for all key questions and not only when the p-value is less than 0.05.

Please outline where this information can be found within the submission (e.g., sections or figure legends), or explain why this information doesn’t apply to your submission:

Statistical analysis is described in the methods section and specified where test methods differ. Statistical results are in figures and their associated legends, as well as in the associated text.

(For large datasets, or papers with a very large number of statistical tests, you may upload a single table file with tests, Ns, etc., with reference to sections in the manuscript.)

**Group allocation**

- Indicate how samples were allocated into experimental groups (in the case of clinical studies, please specify allocation to treatment method); if randomization was used, please also state if restricted randomization was applied
- Indicate if masking was used during group allocation, data collection and/or data analysis

Please outline where this information can be found within the submission (e.g., sections or figure legends), or explain why this information doesn’t apply to your submission:

Groups were heterozygous mutant or non-mutant littermates, and calbindin expressing or non-expressing cells. In inhibitory immunohistochemical experiments ectopic calbindin expressing principal cells were compared with normotopic calbindin negative cells. For determining principal cell morphological group in figure 6 (comp vs simp), cells were grouped independently by two blinded judges. Cells where judges agreed on the morphological group were included, in <5 cases where there was disagreement the data was not included.

**Additional data files (“source data”)**

- We encourage you to upload relevant additional data files, such as numerical data that are represented as a graph in a figure, or as a summary table
- Where provided, these should be in the most useful format, and they can be uploaded as “Source data” files linked to a main figure or table
- Include model definition files including the full list of parameters used
- Include code used for data analysis (e.g., R, MatLab)
- Avoid stating that data files are “available upon request”

Please indicate the figures or tables for which source data files have been provided:

All source data are provided as Excel spreadsheets. A github can be made available for the source code for principal cell shape clustering.
